# Programmable Anisotropic Hydrogels with Localized Photothermal/Magnetic Responsive Properties

**DOI:** 10.1002/advs.202202173

**Published:** 2022-07-20

**Authors:** Hang Chen, Xiaoyuan Zhang, Li Shang, Zhiqiang Su

**Affiliations:** ^1^ State Key Laboratory of Chemical Resource Engineering Beijing Key Laboratory of Advanced Functional Polymer Composites Beijing University of Chemical Technology Beijing 100029 P. R. China; ^2^ Key Laboratory of Bio‐Inspired Smart Interfacial Science and Technology of Ministry of Education School of Chemistry Beihang University Beijing 100191 P. R. China; ^3^ State Key Laboratory of Solidification Processing School of Materials Science and Engineering Northwestern Polytechnical University and Shaanxi Joint Laboratory of Graphene (NPU) Xi'an 710072 P. R. China

**Keywords:** 3D printing, data storage, hydrogels, MoS_2_ nanosheets, smart materials

## Abstract

Programmable smart materials that can respond locally to specific stimuli hold great potential for many applications, but controllable fabrication of these materials remains challenging. This work reports the development of novel programmable anisotropic materials with both magnetic and photothermal stimuli‐responsiveness, which are fabricated by anchoring thermosensitive poly(*N*‐isopropyl acrylamide) (PNIPAm) and magnetic Fe_3_O_4_ nanoparticles on the surface of MoS_2_ nanosheets. Further embedding PNIPAm‐MoS_2_/Fe_3_O_4_ into 3D‐printed hydrogel cubes results in stimuli‐responsive building blocks, and the magnetic field can precisely control their orientation and near‐infrared (NIR) light absorbing property. Particularly, the variation of the orientation of MoS_2_/Fe_3_O_4_ block results in obvious changes of their photothermal efficiency and optical property. By exploiting the anisotropy of MoS_2_/Fe_3_O_4_ and their NIR light responsiveness, thermally‐induced phase transitions in individual 3D printed hydrogel building block can be locally controlled for magnetic field‐assisted programming a quick response (QR) code. Alternatively, fluorescent QR code with high contrast and security level can be achieved by photothermal‐induced release of fluorescent dyes. These 3D printed magnetically programmed hydrogels hold great potential for application in information storage, intelligent materials, and precise therapy.

## Graphical abstract

Magnetic‐oriented and photothermal‐responsive smart materials have been fabricated by anchoring thermosensitive polymers and magnetic nanoparticles on the surface of MoS_2_ nanosheets. Thermoresponsive and optical properties of MoS_2_/Fe_3_O_4_‐based hydrogels can be locally programmed and modulated via 3D magnetic printing, which make them promising for application in fields such as information storage, intelligent materials, and precise therapy.

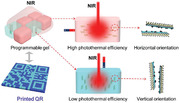

## 1 Introduction

In nature, many amazing composite materials, such as abalone shell, peacock mantis shrimp, and mammalian cortical bone, possess distinct hierarchical structures, where the internal orientation of fillers can optimally accommodate external stimulus such as light, humidity, and temperature.^[^ ^1^ ^]^ These natural phenomena have provided scientists inspiring ideas for the design and synthesis of novel engineered materials via controlling the orientation of building blocks inside the materials.^[^ ^2^ ^]^ For example, studies showed that orientated fibers in the polymer matrix could significantly enhance the composites’ mechanical properties in an anisotropic way.^[^ ^3^ ^]^ Therefore, scientists have made great efforts in recent years to develop advanced biomimetic materials inspired by the orientation‐enhanced features of natural systems.^[^ ^4^ ^]^

The fabrication of smart multifunctional materials, which can respond to distinct stimuli, such as light, heat, electric, and magnetic fields,^[^ ^5^ ^]^ strongly depends on the rational integration of different stimuli‐responsive motifs. Unfortunately, most reported materials can only display one specific orientation as a whole under the force field and lack the block‐by‐block programmable capability.^[^ ^3^, ^6^ ^]^ In the present work, we report the fabrication of novel programmable anisotropic materials with magnetic and photothermal dual stimuli‐responsiveness. Smart anisotropic hydrogels have been fabricated by anchoring functional motifs consisting of thermosensitive poly(*N*‐isopropyl acrylamide) (PNIPAm) and magnetic iron oxide nanoparticles (Fe_3_O_4_ NPs) on the surface of 2D molybdenum disulfide (MoS_2_) nanosheets. Herein, the presence of thermosensitive PNIPAm allows the formation of hydrogels that can act as a smart “thermal switch” to spatially display distinct thermo‐responsive colors and regulate the release of loading molecules, while Fe_3_O_4_ NPs enable the sensitive orientation of MoS_2_/Fe_3_O_4_ in specific directions under the magnetic stimulus. MoS_2_ nanosheets, an emerging type of 2D materials, exhibit significant advantages such as high specific surface area, excellent biocompatibility, easy modification, and ultrahigh photothermal conversion efficiency.^[^ ^7^ ^]^

These PNIPAm‐MoS_2_/Fe_3_O_4_‐based smart hydrogels can be strategically tailored into unique blocks by 3D magnetic printing, further harnessing for data storage, such as quick dot‐matrix response (QR) code. Importantly, the magnetically programmed orientation of MoS_2_/Fe_3_O_4_ in the hydrogels allows them to write and read data based on the magnetic and photothermal stimulus.^[^ ^8^ ^]^ We demonstrated two potential applications of these smart MoS_2_/Fe_3_O_4_‐based hydrogels: (1) visualization of QR codes based on orientated magnetic blocks in the programmed dot‐matrix; (2) controlled release of loaded optical dyes based on photothermal effect. In both approaches, consumer mobile devices can be easily integrated to enable real‐time recording and analysis, enabling further used in information storage, intelligent materials, and precise therapy.

## 2 Results and Discussion

**Figure 1:**
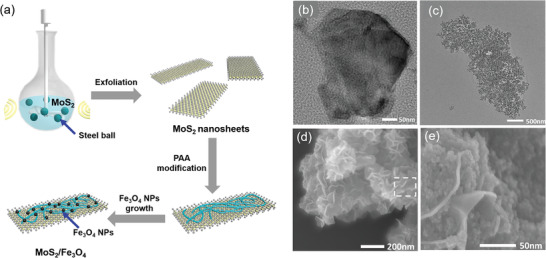
a) Schematic illustration of the synthesis process. Representative TEM images of b) MoS_2_ nanosheets and c) MoS_2_/Fe_3_O_4_. d) Representative SEM images of MoS_2_/Fe_3_O_4_ composites, and e) a close‐up of highlighted area.

In order to achieve sensitive stimulus response of MoS_2_/Fe_3_O_4_‐based hydrogels, it is important to rationally integrate magnetic/thermal dual response motifs in a controllable manner. As illustrated in **Figure** **1**a, our strategy of fabricating MoS_2_/Fe_3_O_4_ composites is rather straightforward. MoS_2_ nanosheets were first prepared by liquid exfoliation of bulk MoS_2_ in the presence of *N*‐methyl pyrrolidone, where a horizontally stirred centrifugal ball mill was adopted to minimize the potential damage of the rigid collision to the exfoliated nanosheets. Transmission electron microscopy (TEM) images showed that the synthesized MoS_2_ nanosheets appear relatively uniform, with only a tiny number of kinks and a narrow size distribution of 0.4–0.8 µm (Figure 1b). High‐resolution TEM (HRTEM) imaging indicates that the crystal lattice fringes with a *d*‐spacing of 0.280 nm can be assigned to the (100) plane of hexagonal MoS_2_ (Figure S1a,c, Supporting Information),^[^ ^9^ ^]^ which is consistent with the 2H‐MoS_2_ (PDF card No. 37–1492). Atomic force microscopy (AFM) images further revealed the flat nature of the MoS_2_ nanosheet surface, with a thickness of 1.8 ± 0.2 nm (Figure S1e, Supporting Information). Subsequently, in the presence of the ammonium persulphate (APS) initiator, the acrylic acid (AA) monomers were polymerized on the nanosheet surface in the form of a wall‐like polymer structure (Figure 1d,e, Supporting Information). Polyacrylic acid (PAA)‐modified MoS_2_ nanosheets can provide abundant anchoring sites for further conjugating Fe^3+^ via coordinating with the carboxyl groups. Consequently, Fe_3_O_4_ NPs can be formed on the surface of MoS_2_ nanosheets through coprecipitation of Fe^3+^ and Fe^2+^ ions under alkaline conditions.^[^ ^10^ ^]^

Figure 1c shows the representative TEM image of obtained MoS_2_/Fe_3_O_4_, and a high density of Fe_3_O_4_ NPs were observed on the surface of MoS_2_ nanosheets. HRTEM images (Figure S1b,d, Supporting Information) further revealed the presence of the crystal lattice fringes with a *d*‐spacing of 0.250 nm, which can be assigned to the (220) plane of Fe_3_O_4_.^[^ ^11^ ^]^ The corresponding AFM images revealed the uniformity of Fe_3_O_4_ NPs on the MoS_2_ surface (Figure S1f, Supporting Information), and the thickness was increased to 5.0 ± 0.4 nm. Furthermore, X‐ray photoelectron spectroscopy (XPS, Figure S2, Supporting Information) and X‐ray diffraction (XRD, Figure S3, Supporting Information) also confirm the successful synthesis of MoS_2_/Fe_3_O_4_. Scanning electron microscopy (SEM) images showed wall‐like structures on the surface of MoS_2_/Fe_3_O_4_ (Figure 1d), which is likely due to the slow polymerization of acrylic acid at low temperatures.^[^ ^12^ ^]^ The magnified SEM image (Figure 1e) of MoS_2_/Fe_3_O_4_ further shows that magnetic Fe_3_O_4_ NPs are also widely distributed among the PAA walls.

**Figure 2:**
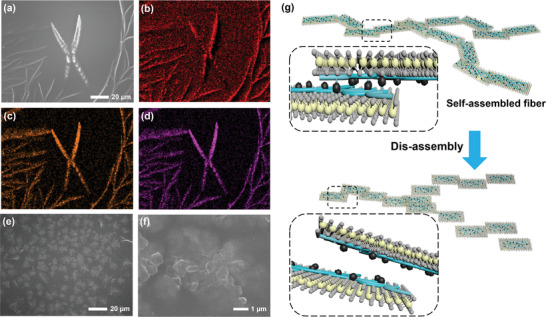
a) Representative SEM image of self‐assembled network‐like fibrils, and elemental mapping of b) C, c) Mo, and d) Fe. SEM images of e) disassembled nanosheets and f) magnified image. g) Schematic illustration of MoS_2_/Fe_3_O_4_‐assembled fibers (upper) and the disassembly process (lower).

These MoS_2_/Fe_3_O_4_ composites exhibit interesting self‐assembly behavior. As shown in **Figure** **2**a, the MoS_2_/Fe_3_O_4_ will self‐assemble into network‐like fibrils in a static environment. The corresponding energy dispersive x‐ray element mapping performed in SEM (Figure 2b–d) indicates the even distribution of C, Mo, and Fe elements in the MoS_2_/Fe_3_O_4_. PAA modification and the decorated Fe_3_O_4_ NPs on MoS_2_ nanosheets are expected to increase the surface area of MoS_2_/Fe_3_O_4_ significantly. Moreover, the nanosheets modified by negatively charged polymers can connect via interactions between carboxyl groups on the surface of different nanosheets and metal ions. As a result, these nanosheets will self‐assemble into nanobelts driven by the surface energy.^[^ ^13^ ^]^ Interestingly, these assembled structures will collapse into separated nanosheets under external perturbations such as magnetic stirring. As shown in Figure 2e,f, after stirring for 30 min, the network‐like MoS_2_/Fe_3_O_4_ fibrils are entirely disintegrated into nanosheets (diameter: ≈1 µm) without the appearance of fibrils in the solution, likely due to the large shear force. Upon further placing disassembled solution at room temperature for 24 h, obvious fibril structures can be observed again (Figure S4, Supporting Information). This interesting observation suggests that the assembly process of MoS_2_/Fe_3_O_4_ is a highly dynamic and reversible process owing to the noncovalent interactions between these magnetic nanosheets. The self‐assembly process is a spontaneous reaction driven by surface interactions, while the disintegration process requires the torque provided by an alternating magnetic field to disassemble the self‐assembly. The outermost layer of Fe_3_O_4_ NPs prepared by the coprecipitation method contains excess amounts of Fe^3+^ ions, which can chelate with the carboxyl groups of different nanosheets. Therefore, MoS_2_/Fe_3_O_4_ can be connected to form nanobelts by magnetic NPs, and the nanoribbons can then form an entangled network structure. As a result, these NPs endowed MoS_2_/Fe_3_O_4_ with high paramagnetism and promoted the ordered aggregation of MoS_2_/Fe_3_O_4_ into network‐like fibrils (Figure 2g).

**Figure 3:**
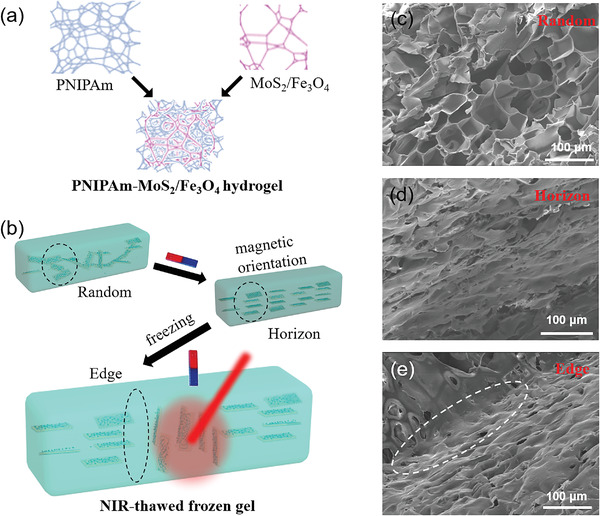
Schematic illustration of a) the fabrication of PNIPAm‐MoS_2_/Fe_3_O_4_ hydrogel and b) programming the orientation of MoS_2_/Fe_3_O_4_ under the magnetic field followed by NIR light‐treatment of frozen hydrogels. Representative SEM images of PNIPAm‐MoS_2_/Fe_3_O_4_ hydrogels under different conditions: c) random distribution without any treatment; d) treatment with horizontal magnetic field; e) NIR light‐thawed frozen gels (the marked area with dotted white line is the edge between the head and middle part).

In order to endow these magnetic MoS_2_/Fe_3_O_4_ with thermo‐responsive features, PNIPAm was then employed to functionalize MoS_2_/Fe_3_O_4_ owing to its good temperature sensitivity and reasonable critical temperature.^[^ ^14^ ^]^ Importantly, these PNIPAm polymers enable the formation of hydrogels, which facilitate their further integration into usable devices. To achieve the anisotropic orientation of MoS_2_/Fe_3_O_4_, it is necessary to ensure that MoS_2_/Fe_3_O_4_ disperse evenly in the hydrogels. As illustrated in **Figure** **3**a, the molecular chain of PNIPAm can form interpenetrating structures with the MoS_2_/Fe_3_O_4_ nanoribbons, resulting in the formation of PNIPAm‐MoS_2_/Fe_3_O_4_ hydrogels. At this stage, magnetic orientation of MoS_2_/Fe_3_O_4_ within the hydrogels is mostly in a random status, which will result in different orientation temperatures under NIR illumination due to the photothermal effect of MoS_2_ nanosheets. To demonstrate the programmable capability of these smart PNIPAm‐MoS_2_/Fe_3_O_4_ hydrogels, we first applied a parallel magnetic field to modulate the orientation of nanosheets into a specific direction artificially. Afterwards, flash‐frozen was conducted to fix the orientation status of MoS_2_/Fe_3_O_4_ within the hydrogels. The angle between the orientation direction of nanosheets and the direction of NIR light will affect the photo‐thermal efficiency. When the angle is vertical, the photothermal efficiency is the highest. NIR light was applied to a specific position, where the photothermal effect of MoS_2_ nanosheets will locally lead to the magnetically‐programmed orientation of MoS_2_/Fe_3_O_4_ returning to a random status. When further applying a magnetic field perpendicular to the hydrogel, the MoS_2_/Fe_3_O_4_ in the defrozen part will be oriented to be perpendicular to the surface (Figure 3b).

Our design was visually confirmed by SEM imaging the hydrogels at different steps. As shown in Figure 3c, in the beginning, MoS_2_/Fe_3_O_4_ within the hydrogels is randomly orientated in a disordered state. Upon applying the magnetic field horizontally and further flash‐frozen treatment, the MoS_2_/Fe_3_O_4_ sheets are oriented parallel to the surface of the hydrogels (Figure 3d), which is significantly different from Figure 3c. After the subsequent NIR illumination and magnetic orientation treatment, one can see the obvious coexistence of two different orientations of nanosheet structures in the edge of the NIR light‐thawed region (Figure 3e). Due to the dual stimuli‐responsive property of MoS_2_/Fe_3_O_4_, their orientation within hydrogels can be precisely controlled by the magnetic field and NIR light.

**Figure 4:**
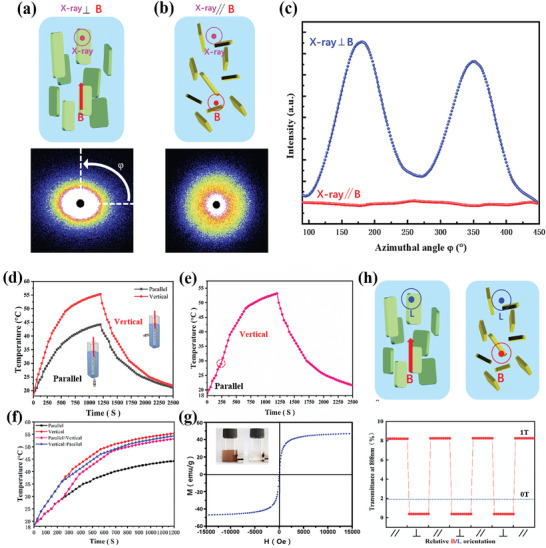
2D SAXS images of magnetic field a) perpendicular and b) parallel to X‐ray. c) Azimuthal angle (*φ*) plots for the 2D SAXS images in (a) and (b). d) Temperature profiles of MoS_2_/Fe_3_O_4_ solution over time by placing magnetic field parallel and vertical to NIR light illumination for the beginning 1200 s. e) Temperature profiles over the time under the dynamic magnetic field from parallel to vertical direction. f) Comparison of temperature profiles of MoS_2_/Fe_3_O_4_ solution under both static and dynamic magnetic fields. NIR light is switched off after 1200 s. g) Magnetization curve of MoS_2_/Fe_3_O_4_ measured at room temperature. h) The transmittance of MoS_2_/Fe_3_O_4_ upon switching the magnetic field (1T) between orthogonal and parallel directions to the incident light. Dotted lines represent the optical transmittance of the dispersion without magnetic flux.

For the quantitative evaluation of the MoS_2_/Fe_3_O_4_ orientation within the hydrogels, the small‐angle X‐ray scattering (SAXS) analysis was performed (**Figure** **4**). After subjecting to the magnetic field that is either perpendicular or parallel to the edge of hydrogels, the frozen gels were cut into small slices (≈1 mm thickness) for the measurement. Upon exposure to an X‐ray beam orthogonal to the applied magnetic field, the MoS_2_/Fe_3_O_4_‐contained hydrogels exhibit an elliptical diffusive pattern that orients its central axis orthogonal to the direction of the magnetic field (Figure 4a). In contrast, the SAXS pattern of X‐ray parallel to the magnetic field direction does not show visible orientation characteristics, as shown in Figure 4b. Further 2D SAXS analysis showed that when the magnetic field is perpendicular to the X‐ray, there are two peaks at an azimuthal angle of 180° and 360° (Figure 4c). However, no peaks were detected in the direction parallel to the X‐ray. Moreover, the orientation of such MoS_2_/Fe_3_O_4_ led to the anisotropy of the gel, which is expected to exhibit significantly different absorption efficiency of NIR light.

As shown in Figure 4d, when the NIR light illuminates the solution of MoS_2_/Fe_3_O_4_ gels, the temperature quickly rises due to the photothermal effect of MoS_2_ nanosheets. However, the photothermal efficiency of MoS_2_/Fe_3_O_4_ is significantly affected by the direction of the magnetic field. Compared with the gels in which the direction of the magnetic field is parallel to the NIR light (denoted as the “parallel” sample), the temperature of the solution with the NIR light vertical to the direction of the magnetic field (denoted as “vertical” sample) was elevated much

faster. Within 2 min of NIR light illumination, the temperature of the “vertical” sample could rise to 55.2 °C, while it only rose to 44.1 °C for the “parallel” sample. Considering that the orientation of MoS_2_/Fe_3_O_4_ is expected to remain certain freedom due to the low crosslinking degree of the gels, we also monitored the gel temperature under the NIR irradiation by varying the direction of the magnetic field. The temperature variations in gels, measured by IR camera, were shown in Figure S5 in the Supporting Information. The temperature in the perpendicular‐oriented gel was obviously higher than that in parallel‐oriented gel. Specifically, under the continuous NIR irradiation, MoS_2_/Fe_3_O_4_ was first set in a parallel magnetic field, then the magnetic field was tuned to the vertical direction at 240 s. The temperature change before and after this process is shown in Figure 4e. It is apparent that the variation of the magnetic field from parallel direction to vertical direction led to an abrupt thermal change in the heating rates, which originates from the difference of their photothermal efficiency. Similar behavior was also observed when the magnetic field direction was changed in reverse (Figure 4f). Magnetization measurements showed that MoS_2_/Fe_3_O_4_ possess a saturation magnetization of 47.6 emu g^−1^, indicating a super‐paramagnetic ability of these MoS_2_/Fe_3_O_4_ at room temperature thanks to the efficient modification of Fe_3_O_4_ NPs (Figure 4g).^[^ ^11^ ^]^ The super‐paramagnetic properties of MoS_2_/Fe_3_O_4_ can effectively prevent the agglomeration of nanosheets after the magnetic field is removed.

Notably, the variation of the orientation of MoS_2_/Fe_3_O_4_ under different magnetic fields also results in remarkable changes in their optical properties. As shown in Figure 4h, when the light source is perpendicular to the magnetic field, the transmittance of MoS_2_/Fe_3_O_4_ hydrogels (slice with 2 mm thickness) was only 0.2%. However, when the light source is parallel to the magnetic field, the transmittance was increased to 8.1%. Meanwhile, the transmittance was 1.8% without any magnetic field, which is reasonable owing to their random orientation status. These observations further confirm that the orientation of MoS_2_/Fe_3_O_4_ in the fabricated hydrogels can be precisely modulated by the magnetic field, which can affect their thermoresponsive and optical properties. Driven by a current‐operated magnetic field, the nanosheet generates angular momentum, which results in torsion and finally stays at the position parallel to the magnetic induction line. With a lateral size of a few micrometers, one would not expect to cause obvious damage to the gel structures. Particularly, the structure of hydrogels is highly flexible with sufficient toleration to the movement of these nanosheets. Importantly, the distinct feature of PNIPAm‐ MoS_2_/Fe_3_O_4_ hydrogels can be locally programmed, making them promising for many applications.

**Figure 5:**
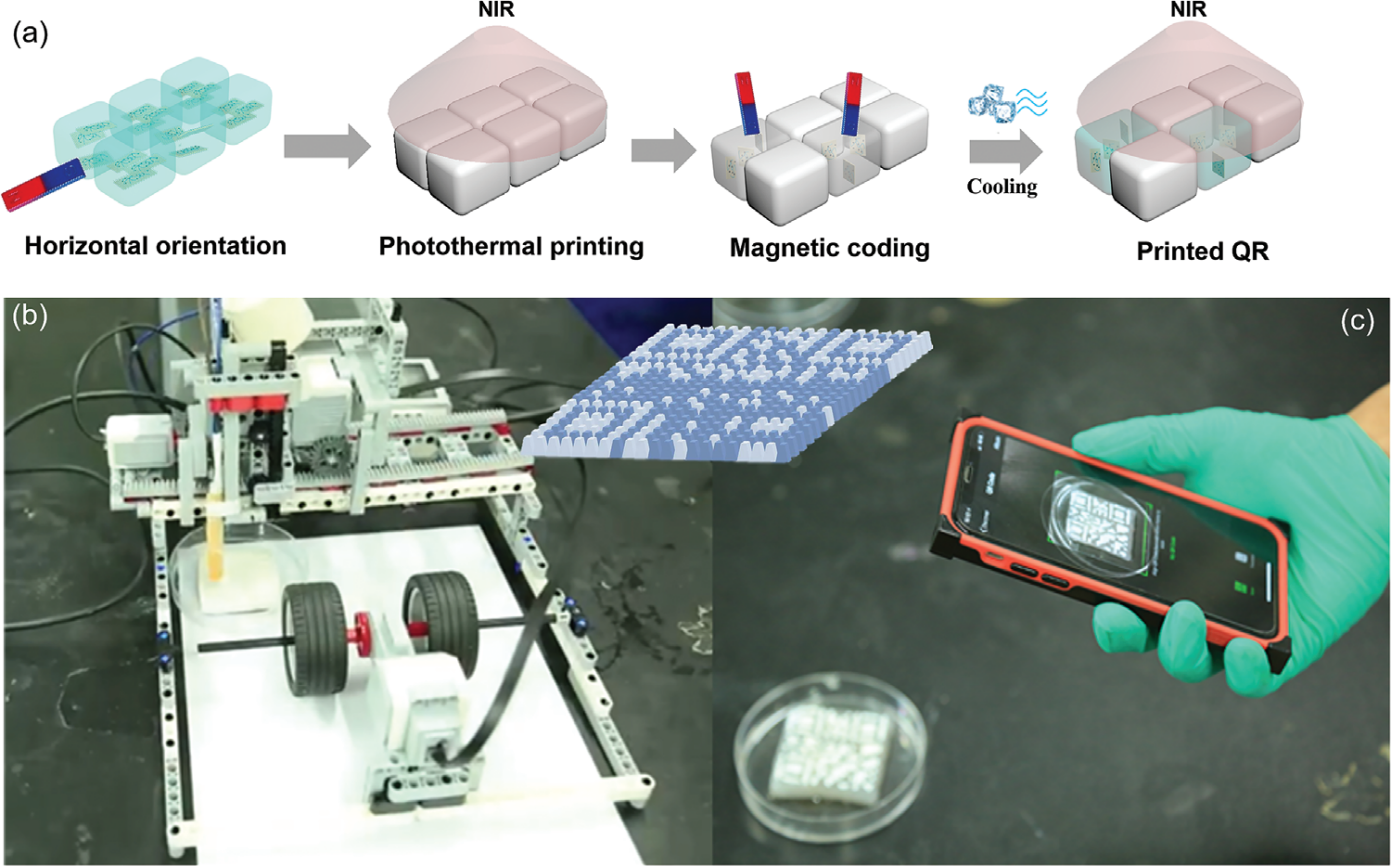
QR recording and reading through photothermal‐triggered color changes of PNIPAm‐MoS_2_/Fe_3_O_4_ hydrogels. a) Schematic illustration of the fabrication strategy. b) Photographs of a 3D magnetic printer for the fabrication of MoS_2_/Fe_3_O_4_‐based QR by using LEGO EV3. c) Formed hydrogel‐based QR code and further reading by smart mobile phone.

Inspired by the 3D magnetic printing technique, programmable LEGO EV3 was used to assemble a 3D magnetic printer.^[^ ^3b^ ^]^ With the activation and withdrawal of the electromagnetic tip, the movement and the electromagnet pulse can be accurately regulated by the printer. After the electromagnetic iron is energized, the nanosheets or nanoparticles are rearranged along the parallel direction of the magnetic induction line. The torque generated by the magnetic field makes the magnetic nanosheet rotate and twist. The QR pattern was transformed into a premade grid of PNIPAm‐MoS_2_/Fe_3_O_4_ hydrogel cubes, in which the orientation of MoS_2_/Fe_3_O_4_ in each cube was predesigned. In this way, the orientation of the MoS_2_/Fe_3_O_4_ in different parts of the QR pattern can be precisely controlled by the LEGO EV3 printer (details can be seen in the Experimental Section). Here, two potential strategies for reading QR codes based on 3D magnetic printing of hybrid hydrogels have been demonstrated. In one method, hydrogels' color change due to NIR irradiation and different MoS_2_/Fe_3_O_4_ orientations was used to develop the magnetically written QR codes (**Figure** **5**a). Firstly, the horizontally‐orientated hydrogels were heated by the NIR light beyond the critical temperature, and the whole hydrogels appeared in white (Figure 5b). The magnetic field was then applied to the programmed positions, allowing the specific MoS_2_/Fe_3_O_4_ block to orient locally perpendicular to the surface. Due to the high photothermal efficiency of horizontally oriented blocks, the programmed positions' color will remain white while these re‐orientated blocks will change to transparent due to low photothermal efficiency (Figure S6, Supporting Information). Subsequently, a consumer‐level mobile phone can be used to read the obtained QR code for further analysis (Figure 5c).

**Figure 6:**
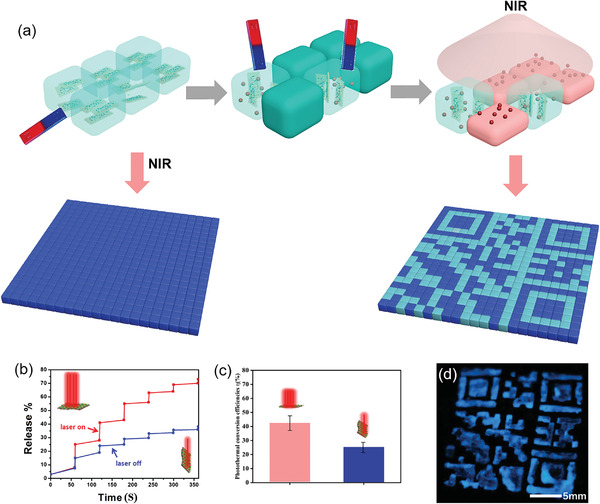
a) Schematic illustration of the fabrication process of fluorescent QR codes based on PNIPAm‐MoS_2_/Fe_3_O_4_ hydrogels. b) Dye release curve over time and c) photothermal conversion efficiency of PNIPAm‐MoS_2_/Fe_3_O_4_ hydrogels under the magnetic field of different directions. d) Photograph of a typical fluorescent QR code under the UV lamp.

Alternatively, the photothermal‐induced release of optical agents from the thermo‐sensitive PNIPAm substrate can be further utilized to generate a fluorescent QR code, as illustrated in **Figure** **6**. Fluorescent molecules, calcium halophosphate phosphor (3Ca_3_(PO_4_)_2_·Ca(F·Cl)_2_:Mn, Sb), were loaded into the PNIPAm‐MoS_2_/Fe_3_O_4_ hydrogels via simple physical encapsulation. The effect of different NIR light directions (related to the orientation of MoS_2_/Fe_3_O_4_) on the release efficiency of fluorescent molecules was then investigated. The release of fluorescent molecules from both vertical and parallel laser directions to MoS_2_/Fe_3_O_4_ was measured by using UV–vis absorption spectroscopy (Figure S7, Supporting Information). The vertical laser to MoS_2_/Fe_3_O_4_ exhibited higher dye release efficiency and release rate than that of the parallel direction, as shown in Figure 6b,c. The release of dyes was terminated when the laser was turned off. It was resumed after the laser was turned on again. The response time of PNIPAm was measured to be 240 s when the sample is 5 mm thick. When NIR laser irradiated the 3D printed hydrogels, the orientated blocks will be selectively heated to create a QR code. As a result, the horizontally oriented MoS_2_/Fe_3_O_4_ exhibit higher photothermal efficiency than other positions (Figure 6a). Due to the volume shrinkage of PNIPAm above the critical temperature, the preloaded fluorescence dyes are released from the specific block of the PNIPAm‐MoS_2_/Fe_3_O_4_. Notably, the grids' substantial gaps provide sufficient thermal insulation between nearby blocks, confining the thermal diffusion within a square area of 1 mm×1 mm. Consequently, a clear fluorescent QR code with high contrast could be formed after washing, as shown in Figure 6d. Compared with the QR‐reading strategy above, this fluorescent coding approach provides better contrast and higher security levels because the codes can only be read upon UV light illumination. Thus, they can be potentially employed in developing advanced anti‐counterfeiting and data storage techniques.^[^ ^15^ ^]^

## 3 Conclusion

In summary, we have developed novel reprogrammable MoS_2_/Fe_3_O_4_‐based smart hydrogels that can respond to both magnetic and photothermal stimuli. The orientation of MoS_2_/Fe_3_O_4_ in hydrogels can be modulated by the direction of the magnetic field, which can regulate the photothermal efficiency of MoS_2_ nanosheets. By further 3D magnetic printing thermosensitive PNIPAm‐grafted MoS_2_/Fe_3_O_4_, we demonstrated the feasibility of photomodulating individual MoS_2_/Fe_3_O_4_ blocks' orientation and optical properties in the hydrogels. These distinct properties make the present MoS_2_/Fe_3_O_4_‐based hydrogels attractive for application in information storage, intelligent materials, and precise therapy. Moreover, this work paves a new avenue for programming the properties of 3D magnetic‐printed functional materials in a high spatial resolution and more controllable manner.

## 4 Experimental Section

### Materials

MoS_2_ powder (less than 2 µm, purity: 99%), APS (purity > 98%), ferric chloride hexahydrate (purity > 99%), ferrous chloride tetrahydrate (purity > 99%), methylene‐bis‐acrylamide (BIS, purity > 99.5%), and polyvinyl alcohol (average molecular weight: 89–98 KD, purity > 99%) were bought from Sigma‐Aldrich. *N*‐methyl pyrrolidone (NMP, 99%), 2, 2′‐azoisobutyronitrile (AIBN, 99%), isopropanol (99.5%), Calcium halophosphate photofluorescent powder 3Ca_3_(PO_4_)_2_·Ca(F·Cl)_2_:Mn, Sb, 99.5%), acrylic acid (99.5%, stabilized with ≈200 ppm 4‐methoxyphenyl), and *N*‐isopropyl acrylamide monomer (stabilized with 500 ppm MEHQ, 99%) were provided by the J&K company. Gel toughening agent, polyvinyl alcohol, was added to PNIPAm as needed. The magnetic printing device was built with the LEGO Mindstorms EV3 programming robot (product no. 31 313).

### Synthesis of MoS_2_ Nanosheets

MoS_2_ nanosheets were prepared by using a highly efficient liquid‐phase exfoliation approach. The solvent‐filled flask was used to combine steel ball milling and stirring for a gentle exfoliation, which resulted in MoS_2_ sheets with better uniformity than the standard ball milling. Moreover, in this method, ultrasonication can assist exfoliation during ball‐milling while the NMP solvent intercalates. The ball milling time was about 4 h, the stirring speed was 300 revolutions per minute, and the steel balls’ diameter was 5 mm. The samples were dried in a vacuum oven at 95 °C for 12 h to remove NMP. The resulting exfoliated MoS_2_ powder was kept at 4 °C before further processing.

### Synthesis of MoS_2_/Fe_3_O_4_

To retain the supernatant, the exfoliated MoS_2_ powder (4 mg) was transferred to an ethanol solution and centrifuged at 4000 rpm. 10 mL of the supernatant was stirred at 2000 rpm, and 0.12 g of APS initiator was added and kept at 25 °C for 30 min. 3 mL of acrylic acid was added dropwise within 10 min and rapidly transferred into the water at 50 °C. 0.410 g of FeCl_3_ in 10 mL water was added to the flask and stirred at 1600 rpm for 30 min to allow the carboxyl groups on the sheet to capture the ferric ions. 0.265 g of FeCl_2_ (with crystal water) was dissolved in 10 mL water and then transferred to the above flask under a nitrogen atmosphere. 2 g of ammonia water was added dropwise under stirring at 1600 rpm. The ferric ions, which are chelated on the sheets' surface, were used to generate magnetic particles. After 60 min of reaction, the precipitate was collected and washed repeatedly with ethanol and water. The obtained magnetic nanosheets can self‐assemble into fibrils.

### Fabrication of PNIPAm‐MoS_2_/Fe_3_O_4_ Hydrogels

Since MoS_2_/Fe_3_O_4_ can self‐assemble into network‐like fibrils, PNIPAm was directly used for generating the hydrogels. 20 mL of MoS_2_/Fe_3_O_4_ solution was transferred to a flask and stirred at 150 rpm to prevent self‐assembly. 1.65 g of NIPAM monomer was added and initiated by AIBN (20 mg) in ethanol at a reaction temperature below 25 °C. Depending on the mold, polyvinyl alcohol (PVA, molecular weight: 13 000, 3%) can be optionally added as a gel toughening agent. The main function of PVA is to provide hydrogen bond for the hydrogel, and to increase the toughness of the gel. As measured by the stress–strain curve, these hydrogels possess a Youngs modulus of ≈4.6 MPa (Figure S8, Supporting Information), suggesting a reasonable mechanical property. Bis (0.0165 g, 1%) was used as a crosslinking agent. The solution was transferred to the mold under the catalysis of *N*′′*N*′′‐tetramethylethylenediamine for 60 s to obtain gels with different shapes.

### Magnetic Printing with LEGO EV3 Printer

A 40 mm×40 mm×4 mm smart gel was taken and cut into a 21×21 grid shape in the 40 mm×40 mm center. Each voxel has a length and width of 1.5 mm. The hydrogels were oriented for 24 h in a horizontal magnetic field environment. The robotic product EV3 supplied by LEGO was assembled into a magnetic printer, which comprises a positioning unit and an electromagnetic tip that can be lowered and activated over a specific grid position by the robot. The QR code in a 21×21 grid was marked in the .wtf format document, and different color parts were distinguished by 0 and 1, respectively, as indicated in Figure S9 in the Supporting Information. The gear line speed's minimum moving unit was adjusted to be 1.5 mm (moving 1 s with an interval of 10 s). The probe was tested for 7 s after the movement was stopped. The electromagnet was set to energize when the magnet probe was down. The electromagnet was de‐energized when retracting. The gel's top‐left mesh was placed at the beginning of the probe to start magnetic orientation printing. Finally, a smart gel with a 2D code array orientation was obtained. The electromagnet can produce a magnetic field of 1 T, ensuring that each unit's gel is oriented for 10 s.

### Fabrication of Fluorescent QR Code

5 g of magnetically oriented printed dehydrated hydrogel was taken and incubated at 70 °C for 2 h. 50 µg of calcium halophosphate phosphor was added to 1 mL water. Then hydrogel was immersed in the solution at 4 °C for 24 h. The gel surface was wiped clean to remove loosely‐adsorbed dyes before further subjected to NIR irradiation (laser wavelength: 808 nm, power: 2.5 W cm^−2^). The integrity of the 2D code pattern was detected with an ultraviolet lamp. For the control group, NIR irradiation and release of the fluorescent dye in the supernatant at 25 °C were also recorded after 0 and 1200 s. All dye encapsulation and release experiments were performed three times for statistical analysis.

### Characterization of MoS_2_/Fe_3_O_4_

The surface coverage of Fe_3_O_4_ was calculated to be (43±2)% based on TEM images. The coverage rate of surface polymers in MoS_2_/Fe_3_O_4_ can be calculated by comparing the coverage rate of Fe_3_O_4_. SEM elemental analysis was employed to determine the ratio of Fe, C, and O elements (Figure S10, Supporting Information), which shows that PAA surface coverage is (37±2)%. The molecular weight of PAA coated on the surface of MoS_2_/Fe_3_O_4_ is related to the temperature. As the temperature increases, the molecular weight of the surface coating polymer increases (Figure S11, Supporting Information).

### Characterization of Gel Modulus and Orientation

The gel modulus can be adjusted by the reaction time. The modulus determines the orientation degree of MoS_2_/Fe_3_O_4_ in the gel (Figure S12, Supporting Information). The light transmittance reflects the degree of orientation of the gel. The sample's thickness was controlled at 5 mm, and the orientation was carried out under a magnetic field strength of 1T. The results showed that a modulus range of 800–1200 Pa is favorable to the orientation of MoS_2_/Fe_3_O_4_ in hydrogels under the magnetic field. A low modulus molecular network cannot fix the MoS_2_/Fe_3_O_4_ orientation. When the gel modulus is greater than 1200 Pa, the orientation of MoS_2_/Fe_3_O_4_ will be firmly fixed by the polymer network.

## Conflict of Interest

The authors declare no conflict of interest.

## Authors Contribution

H.C. performed the experiment and data analysis and wrote the first draft of the manuscript; X.Y.Z. performed data analysis and reviewed the manuscript; L.S. performed data analysis and finalized the manuscript; Z.Q.S. conceived the study, performed data analysis, and reviewed the manuscript.

## Supporting information

Supporting Information

## Data Availability

The data that support the findings of this study are available from the corresponding author upon reasonable request.
