# Supplementary material for: Programmable Anisotropic Hydrogels with Localized Photothermal/Magnetic Responsive Properties
Source: Adv Sci (Weinh). 2022 Jul 20;9(26):2202173. doi: 10.1002/advs.202202173 (PMC9475551; doi:10.1002/advs.202202173)
Supplement: Supplementary file 1 — Supporting Information [file ADVS-9-2202173-s001.pdf]

Supplementary Information

Programmable Anisotropic Hydrogels with Localized Photothermal/Magnetic Responsive Properties

Hang Chen[a,b], Xiaoyuan Zhang[a] , Li Shang[c,], and Zhiqiang Su[a,*]*

[a]*State Key Laboratory of Chemical Resource Engineering, Beijing Key Laboratory of Advanced Functional Polymer Composites, Beijing University of Chemical Technology, Beijing 100029, China*

[b] *Key Laboratory of Bio-Inspired Smart Interfacial Science and Technology of Ministry of Education, School of Chemistry, Beihang University, Beijing 100191, P. R. China*

[c] *State Key Laboratory of Solidification Processing, School of Materials Science and Engineering, Northwestern Polytechnical University and Shaanxi Joint Laboratory of Graphene (NPU), Xi'an, 710072, China*

*Corresponding authors.

E-mail: suzq@mail.buct.edu.cn (Z.Q.S); li.shang@nwpu.edu.cn (L.S.)

Calculation of the photothermal conversion efficiency

The photothermal conversion efficiencies (η) were measured according to the reported method:

$$\eta = \frac{hs(T_{MAX} - T_{Surr}) - Q_{Dis}}{I(1-10^{-A808})} \quad (1)$$

h is the heat transfer coefficient; s is the surface area of the container. Q_{Dis} represents heat dissipated from the laser mediated by the solvent and container. *I* is the laser power and A is the absorbance at 808 nm.

$$hs = \frac{mC_{water}}{\tau s} \quad (2)$$

m is the mass of the solution containing the photoactive material, C is the specific heat capacity of the solution (C_{water} = 4.2 J/(g•°C)), and τs is the associated time constant.

$$t = -\tau s In(\theta) \quad (3)$$

θ is a dimensionless parameter, known as the driving force temperature

$$\theta = \frac{T - T_{Surr}}{(T_{Max} - T_{Surr})} \quad (4)$$

T_{Max} and T_{Surr} are the maximum steady state temperature and the environmental temperature, respectively.

Supplementary Figures

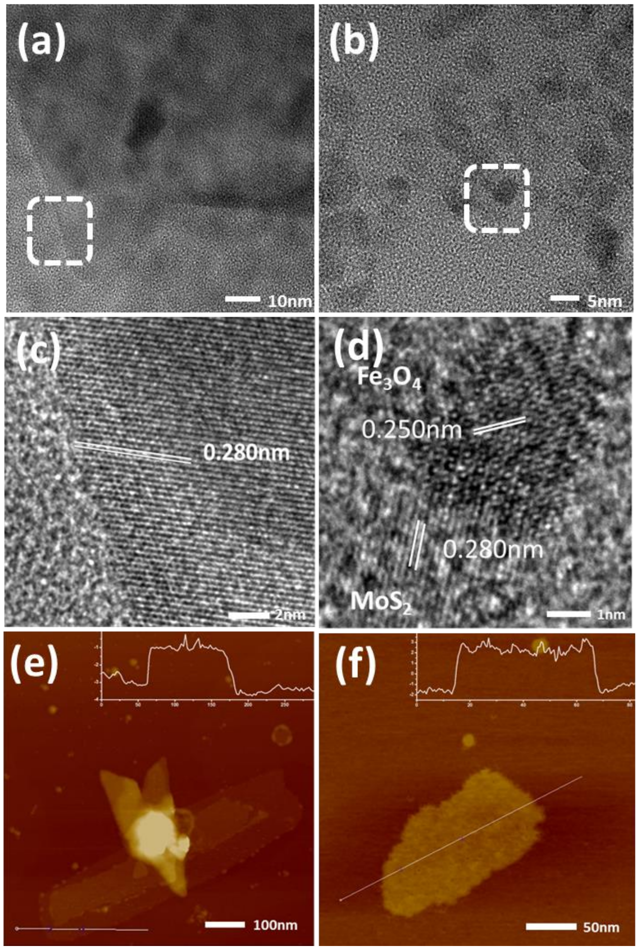

Figure S1. TEM images of (a, c) MoS_2 nanosheets and (b, d) MoS_2/Fe_3O_4. AFM images of (e) MoS_2 nanosheets and (f) MoS_2/Fe_3O_4. The section analysis of MoS_2 nanosheets and MoS_2/Fe_3O_4 indicate the formation of uniform Fe_3O_4 NPs on the surface of MoS_2 nanosheets.

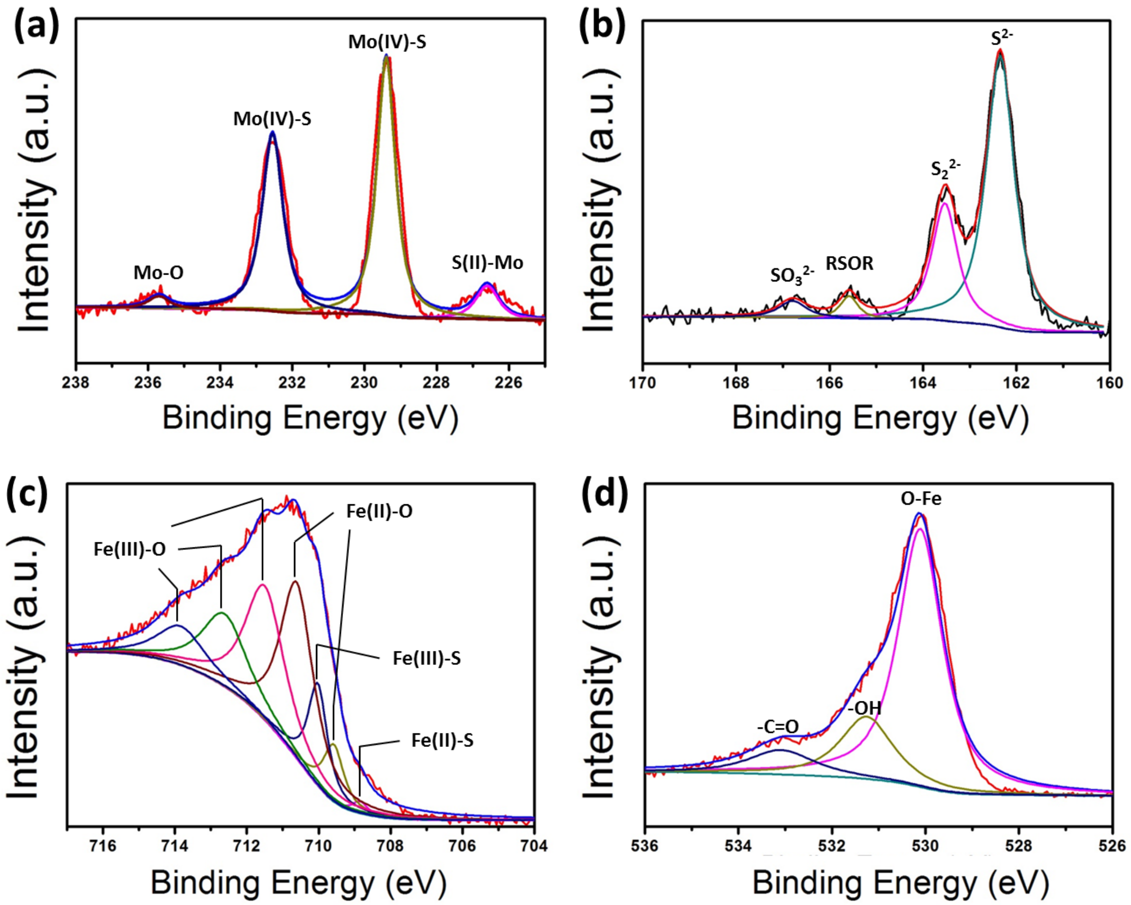

Figure S2. XPS spectra of MoS_2/Fe_3O_4: (a) Mo, (b) S, (c) Fe, and (d) O. In Figure S2a, three peaks at 228.1, 232.0 and 235.1 eV correspond to the $Mo3d_{5/2}$ (Mo^{4+}), $Mo3d_{3/2}$ (Mo^{4+}), and $Mo3d_{5/2}$ (Mo^{6+}), respectively. In Figure S2b, two peaks at 161.0 and 162.0 eV correspond to the $2p_{3/2}$ and $2p_{1/2}$ of divalent sulfide ions (S^{2-}), respectively. A peak at 168.0 eV corresponds to the $2p_{3/2}$ of hexavalent sulfide atom in sulfate (SO_3^{2-}). The peak at 165.6 eV corresponds to the R-OSR. The fitted curves show two peaks at 708.8 and 709.7 eV (Figure S2c), revealing that a new compound composed of Fe and S elements likely formed on the surface of MoS_2/Fe_3O_4. The XPS spectrum of Fe2p with peaks at 711.3, 712.4, and 713.6 eV can be assigned to the Fe^{3+}. Peaks at 709.3 and 710.4 eV are the characteristic peaks of $Fe2p_{3/2}$ in Fe_3O_4, suggesting the coexistence of Fe^{2+} and Fe^{3+}. In Figure S2d, the peak at 529.0 eV can be attributed to the lattice oxygen of Fe_3O_4. The 531.1 eV and 533.0 eV peaks can be assigned to the polyacrylic acid group upon the surface modification.

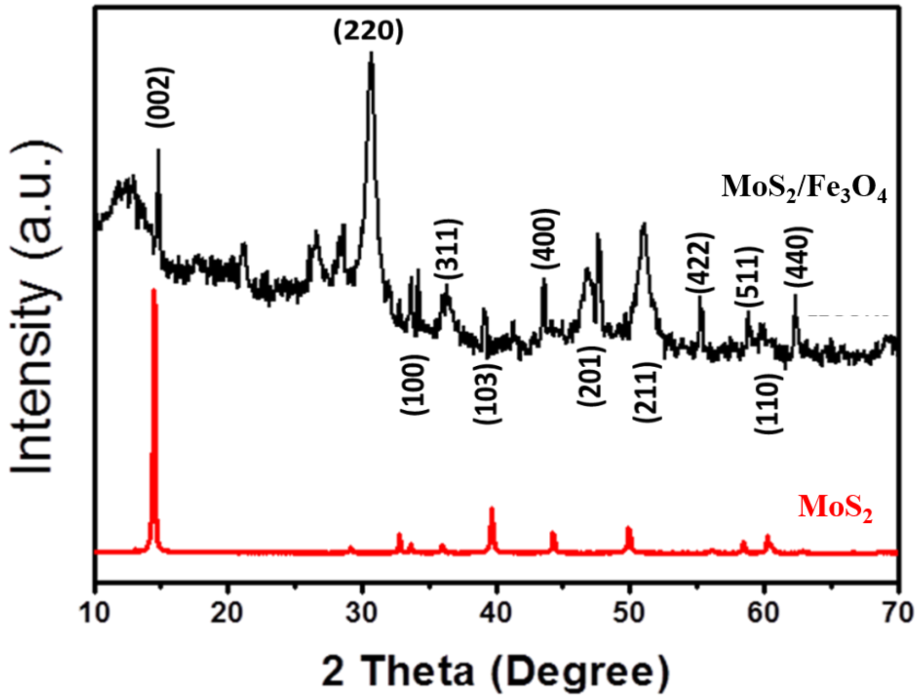

Figure S3. XRD spectra of MoS_2 (red) and MoS_2/Fe_3O_4 (black). XRD analysis shows the corresponding peaks of MoS_2 at (002), (100), (103), (211) and (110). The characteristic crystal plane of Fe_3O_4 appears in the XRD spectrum of MoS_2/Fe_3O_4: (220), (311), (400), (422), (511) and (440). Polyacrylic acid's characteristic peak is at 47° corresponding to (201) crystal plane. This result further confirms the composition of MoS_2/Fe_3O_4 as expected.

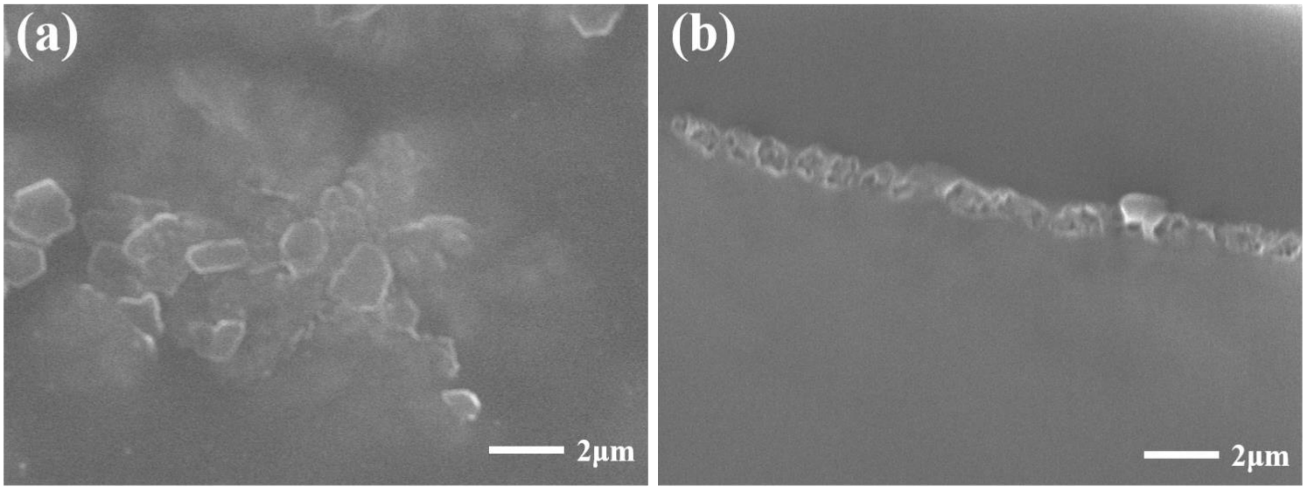

Figure S4 Representative SEM images of MoS_2/Fe_3O_4 after stirring for 30 min (a), and disassembled MoS_2/Fe_3O_4 solution after staying undisturbed for 24 h (b).

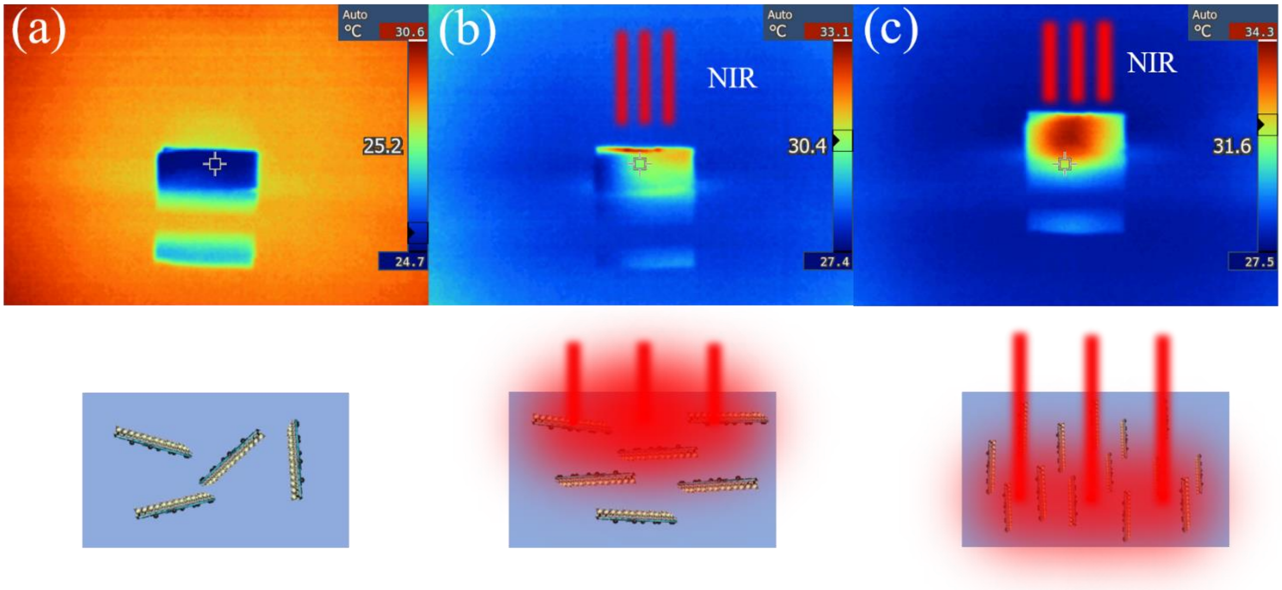

Figure S5. Thermal images of different gels upon irradiated by NIR light, taken by the NIR camera: (a) Non-oriented gel at room temperature; (b) Gel with the orientation perpendicular to NIR light; (c) Gel with the orientation parallel to NIR light.

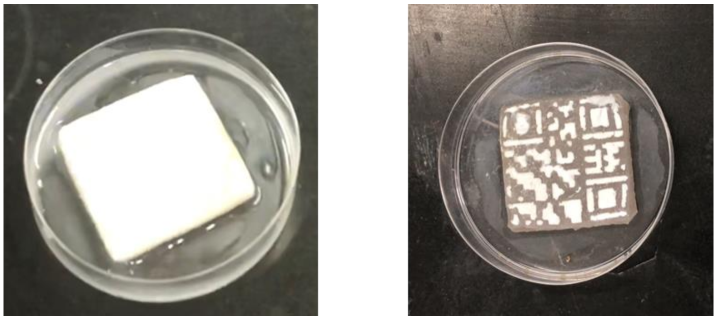

Figure S6. Images of 2-D codes before (left) and after (right) the treatment of magnetic coding and cooling, which clearly shows the change of white gels to designed 2-D patterns.

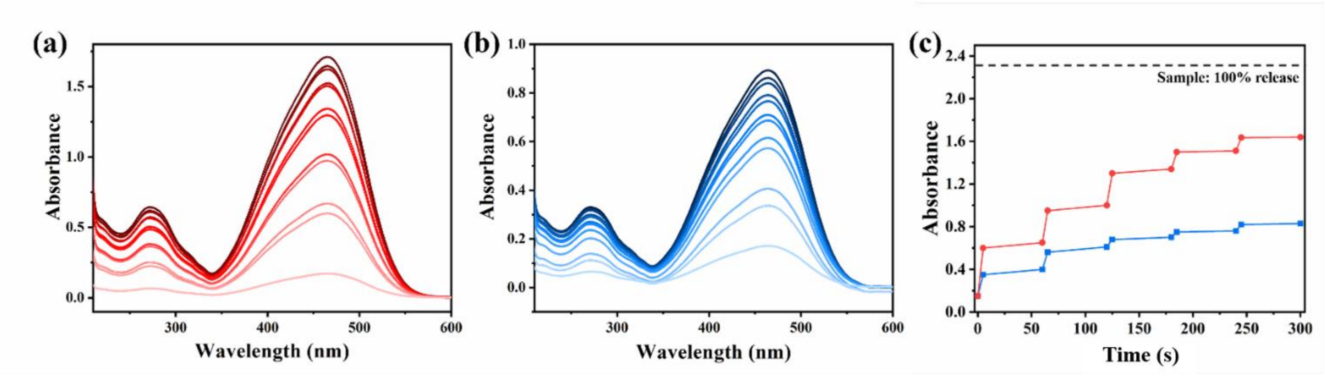

Figure S7. Time-lapse UV-vis absorption spectra of PNIPAm-MoS_2/Fe_3O_4 hydrogels irradiated by (a) vertical and (b) parallel laser. (c) Comparison of the absorbance at 450 nm versus the light irradiation time in (a) and (b). To ensure the consistency of the sample, we evenly divided the gel into two portions. Two parts of the gel were placed under the same NIR laser, and the amount of dyes released was traced by UV-vis absorption spectroscopy.

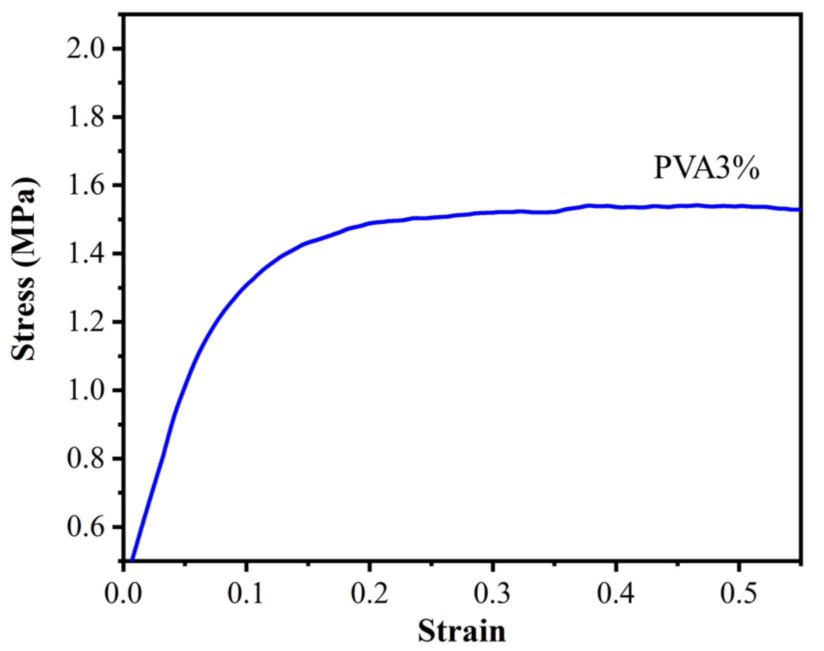

Figure S8. Stress-strain curve of PNIPAm-MoS_2/Fe_3O_4 hydrogels with PVA content of 3%.

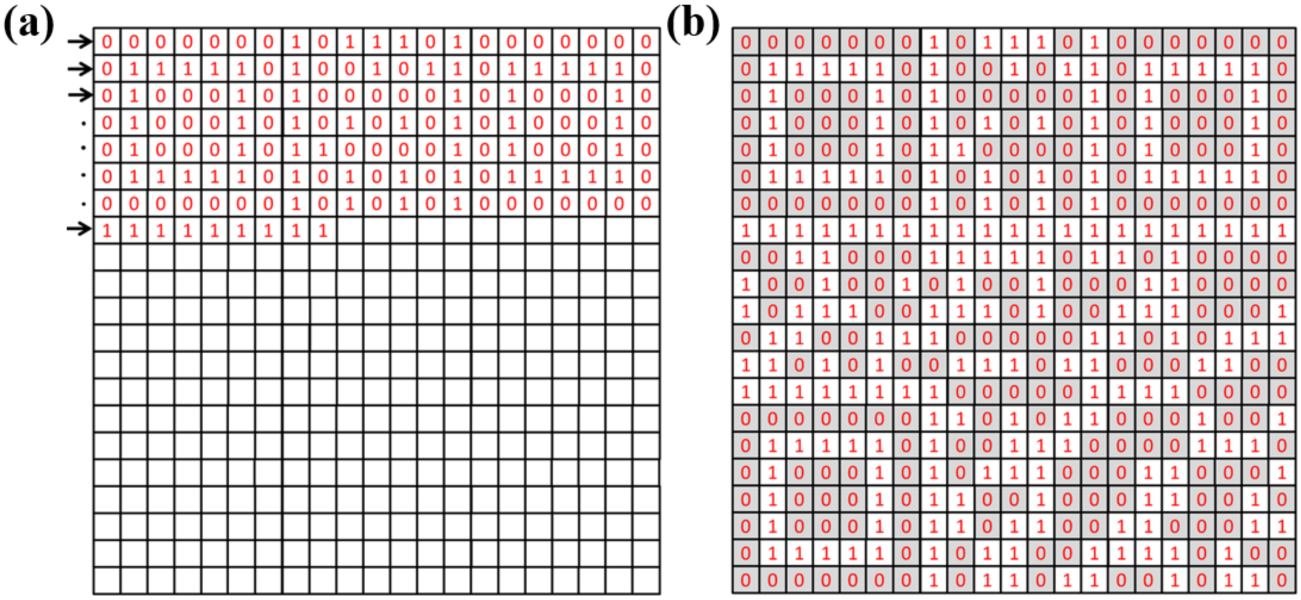

Figure S9. (a) Pre-treatment of gels to 21×21 grid. The direction of the arrow is the printing direction—line by line from left to right. The number “1” represents the lower needle, and the number “0” represents the skipped position. (b) is the complete print pattern.

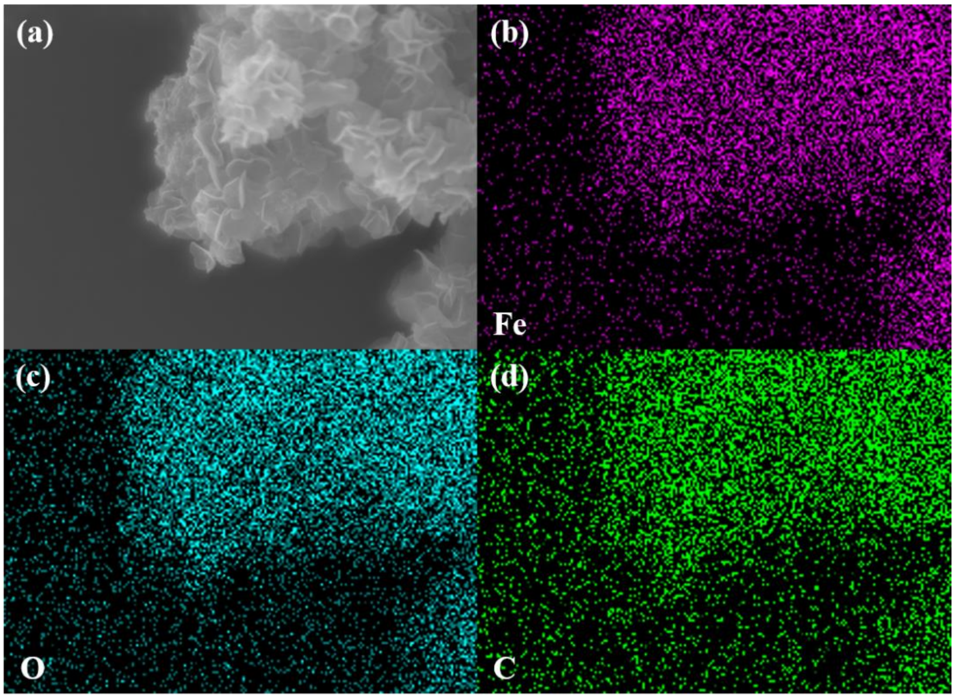

Figure S10. a) SEM image of MoS_2/Fe_3O_4, and the elemental mapping of different elements: (b) Fe, (c) O, and (d) C.

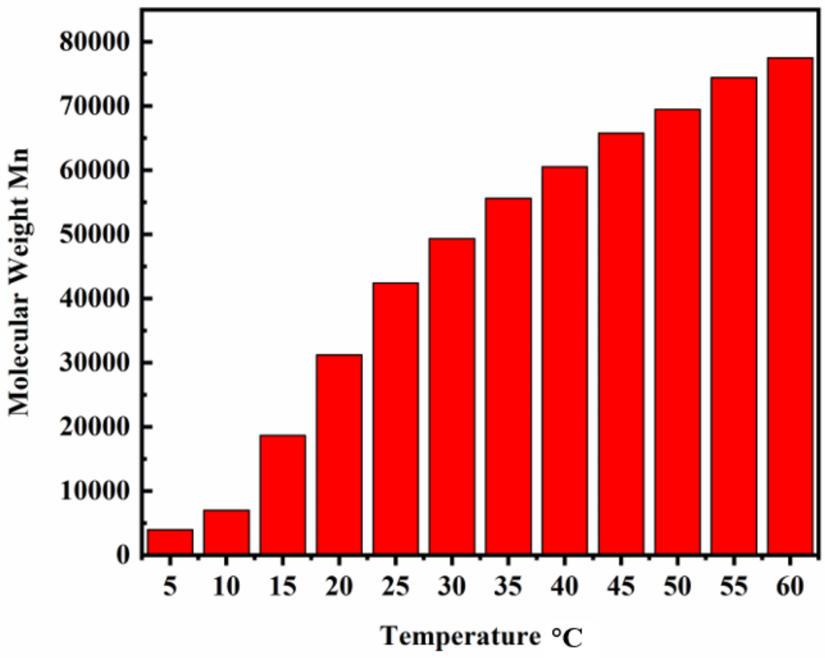

Figure S11. Molecular weight of PAA on the surface of MoS_2/Fe_3O_4 obtained at different reaction temperatures, measured by Agilent Technologies HPLC.

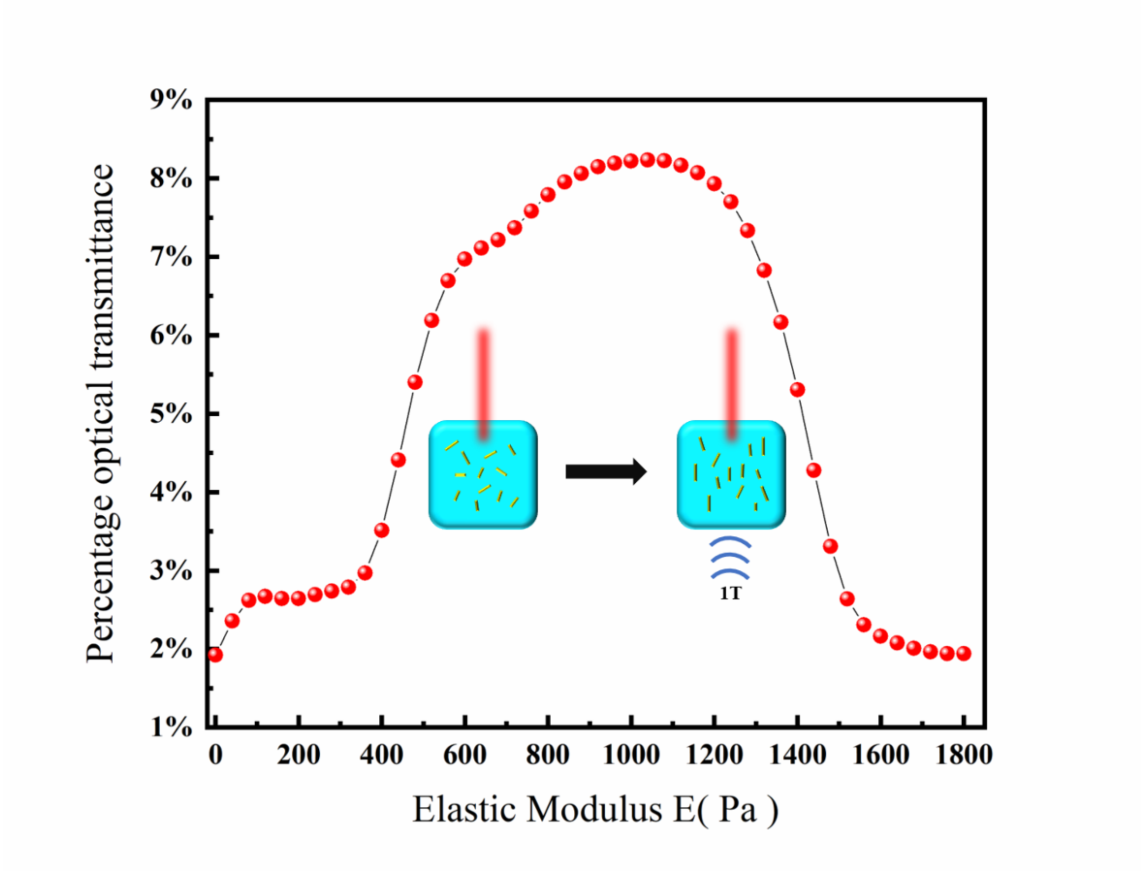

Figure S12. Optical transmittance of PNIPAm- MoS_2/Fe_3O_4 hydrogels versus the gel modulus under 1T magnetic field orientation.
